# Engineering an Antibody V Gene-Selective Vaccine

**DOI:** 10.3389/fimmu.2021.730471

**Published:** 2021-09-09

**Authors:** Larance Ronsard, Ashraf S. Yousif, Julianne Peabody, Vintus Okonkwo, Pascal Devant, Alemu Tekewe Mogus, Ralston M. Barnes, Daniel Rohrer, Nils Lonberg, David Peabody, Bryce Chackerian, Daniel Lingwood

**Affiliations:** ^1^The Ragon Institute of Massachusetts General Hospital, The Massachusetts Institute of Technology and Harvard University, Cambridge, MA, United States; ^2^Department of Molecular Genetics and Microbiology, University of New Mexico School of Medicine, Albuquerque, NM, United States; ^3^Bristol-Myers Squibb, Redwood City, CA, United States

**Keywords:** antibody, B cell receptor, lineage, VLP, prime

## Abstract

The ligand-binding surface of the B cell receptor (BCR) is formed by encoded and non-encoded antigen complementarity determining regions (CDRs). Genetically reproducible or ‘public’ antibodies can arise when the encoded CDRs play deterministic roles in antigen recognition, notably within human broadly neutralizing antibodies against HIV and influenza virus. We sought to exploit this by engineering virus-like-particle (VLP) vaccines that harbor multivalent affinity against gene-encoded moieties of the BCR antigen binding site. As proof of concept, we deployed a library of RNA bacteriophage VLPs displaying random peptides to identify a multivalent antigen that selectively triggered germline BCRs using the human V_H_ gene IGVH1-2*02. This VLP selectively primed IGHV1-2*02 BCRs that were present within a highly diversified germline antibody repertoire within humanized mice. Our approach thus provides methodology to generate antigens that engage specific BCR configurations of interest, in the absence of structure-based information.

## Introduction

Antibody target specificity originates from the interaction between germline B cell receptor (BCR) and cognate antigen. Each germline BCR displays an antigen binding site formed by six antigen binding loops or complementarity determining regions (CDRs) in which antibody gene-encoded CDRs surround the sequence-variable CDRH3 loops (1–3). CDRH3 hypervariability is generated through stochastic N-junctional diversification, where it accounts for the majority of germline BCR diversity and thus forms the principal source of antigen contact affinity (4–6). However, CDRH3 loops do not always explore the antigenic space equally, which is reflected by immunodominance hierarchies in which low frequency BCR target solutions are unable to compete for selection during subsequent antibody affinity maturation within B cell germinal centers (7, 8). Such immunological subdominance is a hallmark of broadly neutralizing antibody (bnAb) responses against pathogens that defy conventional vaccine approaches, including HIV and influenza virus (8–12). It follows that if bnAb targeting solutions are both rare and reliant on randomly emerging CDRH3 configurations, then vaccine-expansion of the corresponding bnAb response will likely prove difficult.

Despite this problem, some human bnAbs and nAbs show biased usage of the gene-encoded CDRs that normally surround the hypervariable CDRH3 loops (13–30). This suggests ‘public’ or genetically-reproducible foundation for pathway-amplifying normally immunologically recessive but protective humoral output (8, 10). Indeed, we have recently demonstrated that normally subdominant human influenza bnAbs can emerge *via* germline-encoded affinity for the bnAb target, and that this gene-endowed reproducibility in targeting specificity enables vaccine-amplification of immunodominant serum bnAb responses, triggered and expanded by a single rationally designed influenza immunogen (31, 32).

Within the HIV space, VRC01-class bnAbs arise through a similar principle, wherein the human V_H_-gene IGHV1-2*02 is reproducibly deployed to structurally mimic the HIV receptor CD4, enabling exceptionally broad neutralization activity (15, 24). Consequently, much effort has been applied to engineer germline stimulating immunogens to selectively prime and expand this genetically conserved bnAb pathway (33–49), and one candidate has entered a Phase 1 clinical trial (ClinicalTrials.gov, NCT03547245). More broadly, IGHV1-2*02 represents one of the more commonly used human antibody V_H_-genes (50–52) and we have recently demonstrated that this human V_H_-sequence also naturally endows germline repertoire with BCRs that target the conserved saccrolipid core of bacterial LPS, imparting a broad-spectrum extrafollicular response against bloodborne bacteria (53).

Given the wide and overlapping utilities of antibody V_H_-endowed or public B cell responses, we sought to develop a vaccine immunogen that selectively primes human B cell lineages *via* preferential contact to the gene-encoded features of the antigen-binding surface. We hypothesized that affinity for a targeted gene-encoded BCR motif would enable selective vaccine-expansion of human germline B cells bearing this feature. To test this central hypothesis, we applied directed evolution on a virus-like particle (VLP) vaccine platform to identify VLPs with multivalent specificity for germline BCRs displaying the CDRs encoded by IGHV1-2*02. This strategy employed a highly immunogenic RNA bacteriophage VLP platform that has been engineered so that it can display diverse random peptide sequences. Starting with a large library of VLPs, we deployed a series of positive and negative selector antibodies with chimeric CDR displays to identify a VLP that engages the genetically conserved features of IGVH1-2*02 BCRs. We then demonstrated that this reagent selectively expands IGHV1-2*02 BCRs *in vivo*, within a purpose-built humanized mouse vaccine model.

## Materials And Methods

### Construction of VLP Selection Libraries

The expression plasmid pDSP62 was described previously (54, 55). Briefly, this plasmid expresses a single-chain dimer version of the MS2 bacteriophage coat protein. The upstream copy has been ‘codon-juggled’ to allow discrimination of annealing sites by primers during reverse transcription (RT) and PCR steps. The plasmid contains the phage T7 promoter and terminator regions from the pET3d vector, a kanamycin resistance gene, and an M13 origin of replication. Unique *Sal*I and *BamH*I restriction sites have been engineered upstream and downstream of the insert-containing copy of coat protein, for use in cloning during affinity selection. VLPs produced using pDSP62 contain 90 copies of the displayed peptide per VLP.

We previously constructed random peptide plasmid libraries for use in our VLP affinity selection protocol (55). Briefly, oligonucleotides were synthesized with 6, 7, 8, 9, 10, 11, 12, 13, or 15 NNS codons, where N represents an equimolar mixture of all four nucleotides and S is an equal mixture of C and G. The thirty-two possible NNS codons encode all 20 amino acids and only a single stop codon. Using the Kunkel site-directed mutagenesis method and a ssDNA phagemid template, we produced plasmid libraries using the pDSP62 vector backbone. Each of the nine plasmid libraries (encoding inserts of different sizes) was generated independently and consisted of at least 10^10^ individual transformants. Plasmid libraries were purified using Qiafilter maxi kits (Qiagen, Venice CA).

### Production of Recombinant VLP Libraries

Individual plasmid libraries were electroporated into the *E. coli* T7 expression strain C41(DE3) (Lucigen) and grown to mid-log phase in LB media with Kanamycin (50 µg/mL). In order to maintain the high diversity of the plasmid library, the efficiency of transformation was monitored, and only highly efficient transformations (with >10^10^ individual transformants) were used to produce VLP libraries. Coat protein expression was induced by the addition of IPTG (1 mM) for three to five hours and bacteria were collected by centrifugation and the pellet was stored at -20°C overnight. Bacteria were lysed in SCB buffer (50 mM Tris, pH 7.5, 100 mM NaCl) with 10 μg/ml of hen egg lysozyme for 1 hour at 4°C, treated with deoxycholate (at a final concentration of 0.05%) for 30 minutes at 4°C, sonicated, and then treated with 10 units/mL of DN*ase* I for 1 hour at 4°C. Soluble protein (which includes VLPs) was separated from insoluble bacterial debris by centrifugation. Soluble protein was concentrated by precipitation with ammonium sulfate at 70% saturation followed by centrifugation, and then resuspended in SCB. VLPs were purified away from contaminating bacterial proteins by size exclusion chromatography using Sepharose CL-4B resin (Sigma-Aldrich) as previously described (56). Fractions that contained VLPs were identified by agarose gel electrophoresis, pooled, and then re-precipitated by the addition of ammonium sulfate at 70% saturation. Precipitated VLPs were collected by centrifugation, solubilized in SCB buffer and dialyzed in SCB overnight using SnakeSkin Dialysis Tubing with a 10 kDa pore size (Thermo Fisher Scientific). VLP libraries were stored at -20°C.

### Positive and Negative Selector ‘LK’ mAbs

LK mAbs were assembled as human IgG1 and displayed the following chimeric antigen binding sites: 1) LK1= gHgL VRC01 (57) = IGHV1-2*02 + VRC01 CDRH3 and FR4 paired with V_L_ gene-reverted VRC01 LC; 2) LK4 = IGHV1-2*02 + [CR6261 CDRH3 and FR4 (58)] paired with V_L_ gene-reverted CR6261 LC (17); 3) LK5 = IGHV3-23*01 + [VRC01 CDRH3 and FR4 (57)] paired with V_L_ gene-reverted VRC01 LC (57); 4) LK6 = IGHV3-23*01 + [CR6261 CDRH3 and FR4 (58)] with V_L_ gene-reverted CR6261 LC (17).

The HC and LC sequences of the LK mAbs were cloned into pVRC8400 (a plasmid containing the CMV IE Enhancer/Promoter, HTLV-1 R Region and Splice Donor site, and the CMV IE Splice Acceptor site upstream of the open reading frame) and co-transfected in 293F cells (59). After six days of expression, the cultures were centrifuged (2,000 × *g*, 10 min) and after filtration of the supernatant (VacuCap 8/0.2μm filters, Pall Corporation), the sample mixed for one hour with Protein G-Agarose (Pierce, Cat #20398). The resin was then washed (6 column volumes of PBS) and the bound IgG was eluted into 50 mM Tris, pH 8, using 1.5 column volumes of low pH IgG elution buffer (Pierce, Cat#21004). The LK mAbs were then concentrated using Amicon Ultra concentrators (30 kDa cut off) and were further resolved by size exclusion FPLC using a Superdex 200 10/300 column (GE Healthcare) followed by SDS PAGE and staining with GelCode Blue (Thermo Scientific, MA).

### VLP Selections

The selection scheme is shown schematically in **Figure 3A**. Selections were performed by coating ELISA plates (Immulon 2HB; Thermo Fisher Scientific) with a total of 500 ng of antibody (LK1, selection rounds 1 and 2, or LK4, rounds 3 and 4) in 50 µL of PBS (phosphate-buffered saline) overnight at 4°C. The wells were then blocked using PBS/2% bovine serum albumin (BSA) in a total volume of 100 µL for 2 hours at room temperature (~25°C). A mixed library of VLPs was generated by mixing of equal amounts of the nine different VLP libraries displaying random sequence inserts ranging from 6 to 15 amino acids in length. 50 µg of the pooled VLPs were suspended in a total of volume of 50 µl of PBS/1% BSA added to wells and then incubated at room temperature for 2 hours at room temperature. After extensive washing with PBS, bound VLPs were eluted by incubating wells with 50 µl of 0.1M glycine (pH 2.7) for 5 minutes. Eluted VLPs were then brought to neutral pH by addition of 5 µl 1M Tris (pH 9.0).

Reverse transcription (RT) was performed using 8 µl (~15%) of the eluent as template, with 1.25 µM of a primer annealing downstream of the MS2 coat protein sequence (5′-TCAGCGGTGGCAGCAGCCAA-3′) and MMLV-RT (Invitrogen) following the manufacturer’s instructions. The product of this reaction was amplified by PCR using High Fidelity Platinum Taq (Invitrogen) and primers that annealed upstream (5’-CTATGCAGGGGTTGTTGAAG-3’) and downstream 5’-CGGGCTTTGTTAGCAGCCGG-3’) of the portion of coat protein that contained the inserted sequence. The PCR product was purified using the QIAquick PCR Purification Kit (Qiagen), digested using BamHI-HF and SalI-HF (New England Biolabs), and then re-ligated into the pDSP62 expression vector. Ligation reactions were ethanol precipitated and resuspended in 10 µl of nuclease-free water. The entire volume was used to transform electrocompetent 10G cells (Lucigen); pooled transformants were grown overnight in a 100 ml culture LB media with kanamycin (50 µg/ml). Plasmid DNA was recovered from these pooled cultures by midiprep (Qiagen) and then used to generate VLPs for subsequent rounds of selection, as described above. As is shown in **Figure 2A**, we performed four rounds of selection, alternating between LK1 (rounds 1 and 2) and LK4 (rounds 3 and 4). After the fourth round of selection, individual transformants were isolated and sequenced. A more detailed protocol describing the generation of VLPs displaying random peptides and affinity selections is also available (60).

### ELISAs

Selected VLPs were tested by ELISA to measure binding to the positive selectant antibodies (LK 1 and LK 4) and negative selectors (LK 5 and LK 6). Briefly, 500 ng of VLPs in a total volume of 50 µl were adsorbed to Immulon II HB ELISA plates overnight at 4°C. The wells were blocked from nonspecific binding using 0.5% dry milk in PBS in a 100 µl volume for 2 hours at room temperature. Wells were then incubated with serial dilutions (diluted in PBS/0.5% milk) of the antibodies of interest (LK1, LK4, LK5, or LK6) for 2.5 hours at room temperature. Following extensive washing with PBS, 50 µL of a 1:4000 dilution of horse radish peroxidase (HRP)-conjugated goat anti-human IgG (Jackson Immunoresearch), diluted in PBS/0.5% milk, was added to each well and incubated for 1 hour at room temperature. ELISAs were developed using TMB substrate (50 µL, EMD Millipore), stopped using 50 µL of 1% HCl, and then absorbance was measured at 450 nm using an accuSkan FC plate reader (Fisher Scientific).

### BCR Triggering *In Vitro*

To evaluate V_H_-dependent signaling by VLP selectants, the LK1, LK4, LK5 and LK6 antibodies were expressed as IgM BCRs within a BCR-surface negative Ramos B cell line that enables display of mono-specific IgM BCRs of interest (17, 59). Ectopic BCRs are stably expressed by through lenti-viral mediated delivery of membrane anchored HC and LC sequences (59). This BCR reporter system has been now widely described and deployed as a tool to rank-order candidate immunogens in antigen receptor triggering studies (15, 31, 59, 61–67) and its display of user-defined BCR sequences has also been extensively characterized and validated by deep sequencing (68).

In this study, reporter B cells displaying LK1, LK4, LK5 or LK6 IgM BCRs were evaluated for antigen receptor signaling following incubation with VLP selectants, as per our standard method (59). In these experiments, 1×10^6^ cells displaying monoclonal BCR were resuspended in RPMI media and exposed to 10µg/ml VLP selectant or 0.5 μg/μl anti-IgM F(ab’)_2_ (Southern Biotech). BCR stimulation was measured kinetically by flow cytometry (LSR II, BD) as the ratio of the Ca^2+^ bound/unbound states of the membrane permeable and ratiometric dye Fura Red. For each LK cell line, the ratiometric measurements were made before and after antigen exposure, wherein data was acquired for 300 seconds after stimulation. All the values were normalized to total Ca^2+^ flux capacity, as defined by exposure of the cells to 10 μg/ml ionomycin (59). Downstream analyses of the data were performed using FlowJo software version 9.5.2 (TreeStar).

### Transgenic Mice

The transgenic mice used in this study were of a previously established model in which human antibody V_H_ usage is constrained to user-defined gene segments, while allowing for normal and random recombination with diverse human D and J segments, which generates an antibody CDRH3 repertoire that is similar to humans, both in relation to length distribution and amino acid usage (31, 53). In this study mice constrained to the human V_H_ gene IGHV1-2*02 (31, 53) were used, and were a gift to D.L. from Bristol-Myers Squibb (Redwood City, CA). The animals were maintained within Ragon Institute’s HPPF barrier facility and the experiments were conducted with IACUC approval (MGH protocol 2014N000252). In this study, both male and female animals, aged 6-10 weeks, were used.

### Vaccine-Expansion of IGHV1-2*02 BCRs *In Vivo*

B cell adoptive transfer experiments were performed as described previously (31). Resting naïve IgM B cells from IGHV1-2*02 HC2 mice were purified from spleen (B Cell Isolation Kit, Mitenyi Biotec, Cat#130-095-813) and transferred to recipient CD45.1^+/+^ C57Bl/6 mice by intravenous injection (1x10^6^ B cells per recipient). After 24h, the recipient mice were immunized intraperitoneally with 100µg of VLP-F2 or VLP displaying irrelevant peptide. After eight days, the mice were euthanized and their spleens obtained and processed at 4°C. Single cell suspensions of the splenocytes were generated using gentle grinding in PBS, cell straining (70µm diameter) and treatment with ACK lysis buffer (31). The cells were washed in PBS and then stained with Blue viability dye (0.025 mg/ml, 5 minutes), and then incubated for 1h with a cocktail of the following anti-mouse antibodies: CD3-BV785 (BioLegend Cat#100231); CD19-PerCP-Cy5.5 (BioLegend Cat#152405); CD138-BV421 (BD Biosciences Cat#566289); CD45.1-PE/Cy7 (BioLegend Cat#110729); CD45.2-Alexa 647 (BioLegend Cat#109817). The cells were washed twice, resuspended in PBS and then measured using flow cytometry (5 Laser LSR Fortessa, BD Biosciences). Compensation for antibody staining was performed using AbC Total Compensation Beads (ThermoFisher). Downstream analyses of the data were performed using FlowJo software version 9.5.2 (TreeStar).

### Statistics

Statistical analyses was performed using Prism Graphpad software. The expansion of CD45.2^+/+^ B cells in response to VLP-F2 or VLP displaying irrelevant peptide was compared using Students’ T-test. The sample sizes were n=5 animals per treatment and an alpha level of 0.05 was deployed throughout.

## Results

### Systematic Overview

**Figure 1:**
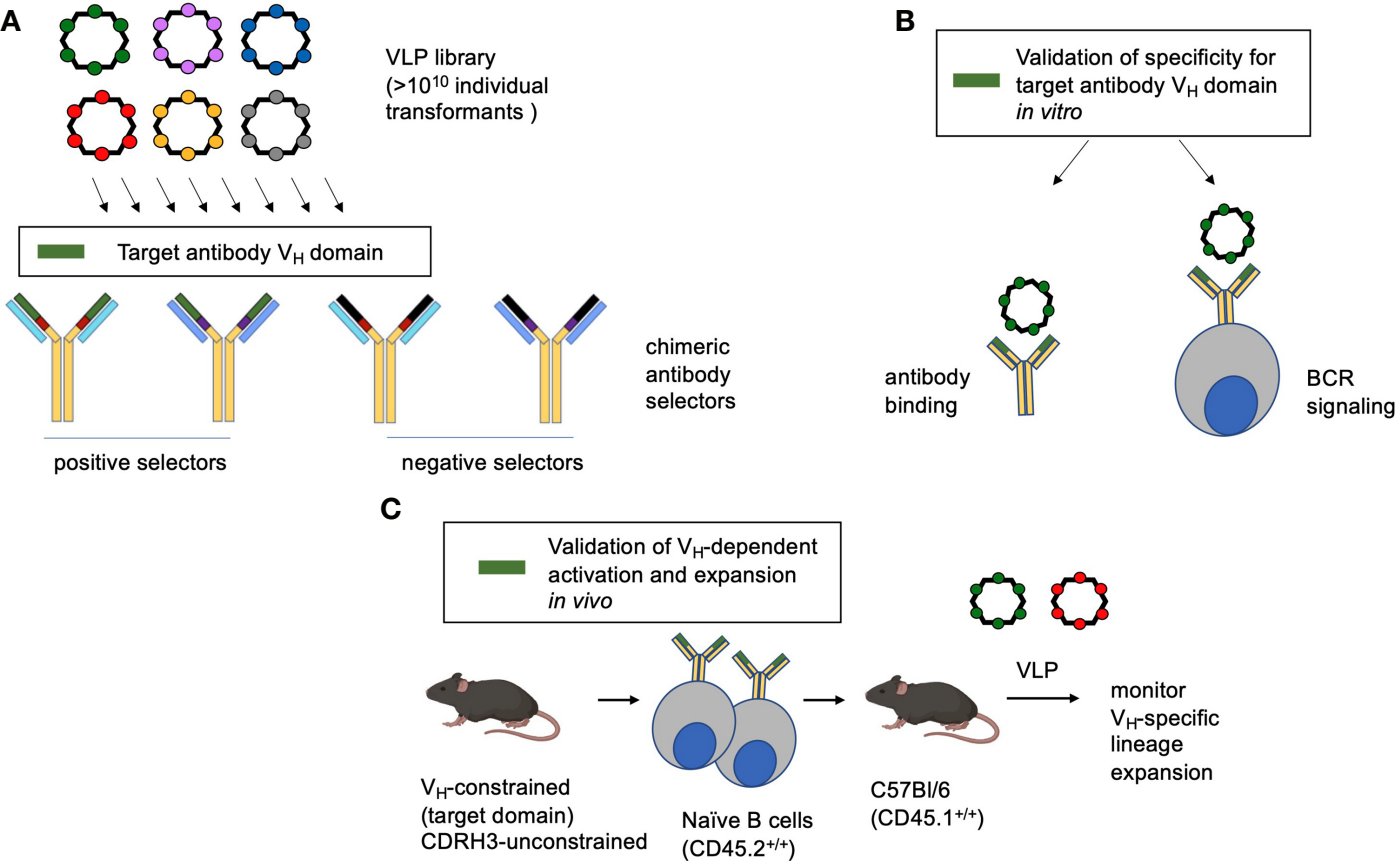
Method overview. **(A)** A highly immunogenic RNA bacteriophage VLP platform has been engineered to display diverse guest peptide sequences. Each VLP encapsidates its own mRNA, meaning that the VLPs can be deployed for affinity selection. The library is then interrogated for specificity to antibodies with chimeric antigen binding sites, serving to identify VLP bearing affinity to a specific antibody feature (in this study the human IGHV1-2*02 domain). **(B)** The identified VLP is then verified by antibody binding and its capacity to trigger the BCR configuration of interest when displayed by a reporter B cell line. **(C)** To evaluate the corresponding B cell lineage expansion *in vivo*, transgenic human-like CD45.2^+/+^ BCRs constrained to the motif are adoptively transferred to recipient CD45.1^+/+^ C57Bl/6 mice. In this case the adoptively transferred B cells are constrained in IGHV1-2*02 usage, but unconstrained all other BCR features and bear human-like diversity in CDRH3 (31, 53).

We present a pipeline for generating multivalent VLP antigens with biochemical specificity to user-defined features of the germline antibody binding site (**Figure 1**). We first apply a highly immunogenic RNA bacteriophage VLP platform that has been engineered so that it can display diverse random peptide sequences (54, 55, 69). Each library contains more than 10^10^ individual transformants, and each individual VLP displays a different guest peptide on its surface and encapsidates its own mRNA, meaning that the VLPs can be deployed for affinity selection. Recombinant antibodies displaying chimeric antigen binding sites are then applied positive and negative selectors to identify VLP with affinity for a particular antibody region of interest, in this study the human IGHV1-2*02 domain (**Figure 1**). The selected VLPs are then evaluated for antibody binding and capacity to elicit the corresponding BCR signaling within a B cell reporter system (59). Finally, the VLPs are assessed for their capability to activate and expand the targeted B cell lineages *in vivo*. In this assay, transgenic mice constrained to the V_H_-sequence of interest [but unconstrained in human-like CDRH3 diversity (31, 53)] are in the CD45.2^+/+^ background and their naïve B cells are adoptively transferred to CD45.1^+/+^ C57Bl/6 mice. The recipient animals are then immunized by the VLPs of interest and the expansion of the CD45.2^+^ B cell lineages is tracked, as in other studies (31, 37–39, 46, 70).

### VLP Library

Previously, we developed a system based on bacteriophage MS2 that combines the affinity-selection capabilities of filamentous phage display with the potent immunogenicity of VLP-platform-based vaccine technologies (54, 55). The coat protein of MS2, a simple icosahedral RNA bacteriophage, was engineered to be highly tolerant of foreign peptide insertions into a surface-exposed ß-hairpin loop structure. Short (6-15 amino acid) random peptide insertions at this site are highly compatible with VLP assembly and are displayed multivalently on the VLP surface (54, 55, 69). VLPs with specific binding characteristics can be affinity selected from large libraries of VLPs displaying random peptides using, for example, mAbs (71, 72) or polyclonal IgG (73, 74). We generated nine independent VLP libraries (displaying random 6-, 7-, 8-, 9-, 10-, 11-, 12-, 13-, & 15-amino acid inserts, respectively) using methods established previously (54, 55).

### Affinity Selections to Identify VLPs That Bind the IGHV1-2*-02 Domain

**Figure 2:**
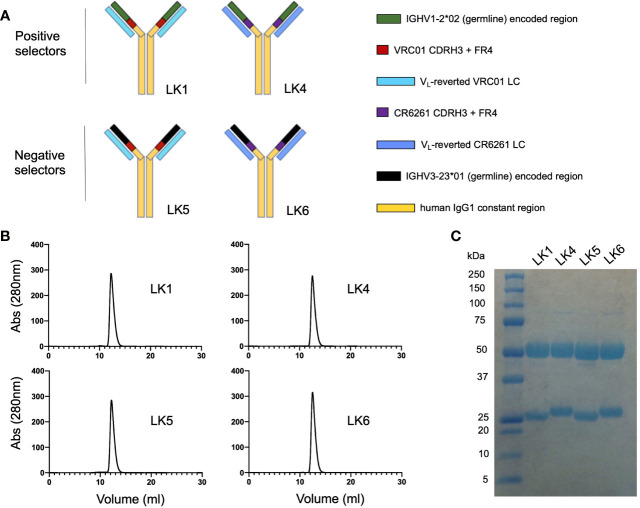
Engineering chimeric antibody loop display for positive and negative selection. **(A)** Chimeric ‘LK’ mAbs were assembled using human IgG1 and displayed chimeric antigen binding sites: 1) LK1= gHgL VRC01 [IGHV1-2*02 + VRC01 CDRH3 and FR4 paired with V_L_ gene-reverted VRC01 LC]; 2) LK4 = IGHV1-2*02 + [CR6261 CDRH3 and FR4] paired with V_L_ gene-reverted CR6261 LC; 3) LK5 = IGHV3-23*01 + [VRC01 CDRH3 and FR4] paired with V_L_ gene-reverted VRC01 LC; 4) LK6 = IGHV3-23*01 + [CR6261 CDRH3 and FR4] with V_L_ gene-reverted CR6261 LC. **(B)** Size exclusion chromatography of the LK mAbs (Superdex 200 10/300 column). **(C)** Purified LK mAbs separated by SDS PAGE and stained with GelCode Blue.

Recombinant selector antibodies displaying chimeric antigen binding sites were referred to as ‘LK’ mAbs (**Figure 2**). Each LK was assembled using IgG1 and displayed the following chimeric paratopes: 1) IGHV1-2*02 + VRC01 CDRH3 and FR4 paired with V_L_ gene-reverted VRC01 LC (=LK1, positive selector); 2) IGHV1-2*02 + CR6261 CDRH3 and FR4 paired with V_L_ gene-reverted CR6261 LC (=LK4, positive selector); IGHV3-23*01 + VRC01 CDRH3 and FR4 paired with V_L_ gene-reverted VRC01 LC (=LK5, negative selector); IGHV3-23*01 + CR6261 CDRH3 and FR4 paired with V_L_ gene-reverted CR6261 LC (=LK6, negative selector). Thus, LK1 and LK4 both share the IGHV1-2*-02 domain, encompassing CDRH1 and CDRH2, but have different CDRH3 domains and light chains (**Figure 2A**). LK5 and LK6 contained IGHV3-23*01, but were otherwise identical to LK1 (LK5) or LK4 (LK6). The LKs were generated recombinantly and proper IgG assembly was confirmed using size exclusion chromatography and SDS PAGE (**Figures 2B, C**). LKs were then applied as positive and negative VLP selectors, where substrate specificity for the IGHV1-2*02 domain could be identified by reactivity to LK1 and LK4 but not to LK5 and LK6.

**Figure 3:**
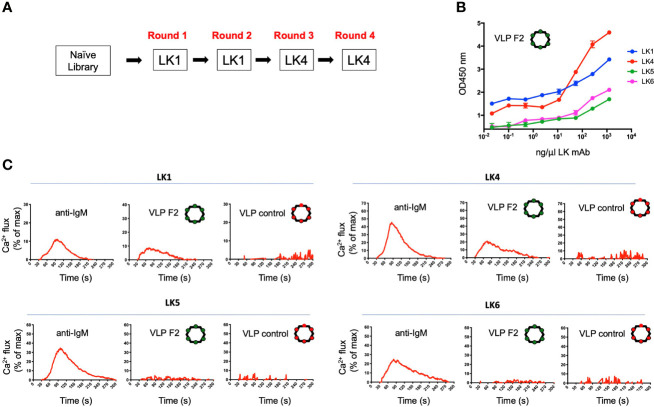
Isolation of a VLP selectant with affinity for BCRs with IGHV1-2*02-encoded domains. **(A)** The VLP library underwent four rounds of positive selection using either LK1 and LK4 mAbs. After each round of selection, the VLPs were screened by ELISA for reactivity to LK1, LK4, LK5 and LK6. **(B)** The VLP-F2 displaying the peptide sequence HMRGGAYATD was isolated which showed affinity for LK1 and LK4 mAbs (mean ± SD, n = 3 replicates), but not LK5 or LK6 mAbs (see other VLP reactivity profiles in **Figure S1**). **(C)** IGHV1-2*02-depedent BCR triggering activity by VLP-F2. To confirm that selective affinity for the IGHV1-2*02 germline domain translated into V_H_-gene-selective BCR activation, the LK antibodies were stably expressed as IgM BCRs in an engineered B cell reporter line that enables monospecific display of user defined BCRs (59). BCR activation was measured kinetically by Ca^2+^ flux using the ratiometric dye Fura Red. Following baseline acquisition (20s), LK BCRs were exposed to anti-IgM, VLP-F2 or VLP-control and the data was acquired for 300s. The baseline reading was then subtracted and the Ca^2+^ flux values were standardized to total cell Ca^2+^ flux capacity as defined by exposure to the ionophore ionomycin. Each run represents the average of two independent fluxes per BCR.

We deployed the LK mAbs to select and screen for VLPs that bound specifically to the IGHV1-2*-02 domain (**Figure 3A**). The VLP library was subjected to four rounds of positive selection, alternating between the LK1 (rounds 1 and 2) and LK4 (rounds 3 and 4) mAbs. Following the final selection, a small number of individual selectants were cloned, sequenced, used to express VLPs, and then tested for binding to the panel of LK antibodies by ELISA. This analysis identified classes of VLPs with different LK binding specificities (**Figure S1**). One VLP (HMRGGAYAYTD; designated F2) bound strongly to both LK1 and LK4, but not LK5 and LK6 (**Figure 3B**), indicating that it was selected for binding to the IGHV1-2*-02 domain. Conceivably, LK5- and LK6- mAbs could have been co-applied as negative selectors to further direct the evolution of VLP selectants toward IGHV1-2*02 domain specificity, however this was not required.

### Selected VLPs Engage and Activate B Cells Expressing IGHV1-2*-02

Multivalent antigen display is a principle that enables BCR crosslinking and receptor signaling (75, 76) and has long been applied to enhance humoral output by vaccines (77–80). As this principle was built into our VLP platform (54, 55), we could test whether VLP-F2 triggered IGHV1-2*02 BCRs. Accordingly, we expressed LK1, LK4, LK5 and LK6 as IgM BCRs in a B cell reporter system (59). In this system, IgM BCRs of interest are stably expressed in Ramos B cells lacking surface display of their endogenous BCRs, enabling evaluation of specific antigen receptor triggering by vaccine candidates *in vitro* (15, 31, 61–68). LK IgM BCR activation was evaluated kinetically, by Ca^2+^ flux, where we found that VLP-F2 triggered signaling from LK1 and LK2 BCRs but not from LK4 or LK5 BCRs (**Figure 3C**). Control VLP failed to stimulate any of the LK BCRs, despite the fact that BCR signaling in response to crosslinking by anti-IgM was comparable across the LK sequences. These data indicated that the VLP-F2 specificity for the IGHV1-2*02-encoded domain translated into IGHV1-2*02-dependent BCR signaling *in vitro*.

### VLP-F2 Selectively Expands IGHV1-2*02 B Cells *In Vivo*

**Figure 4:**
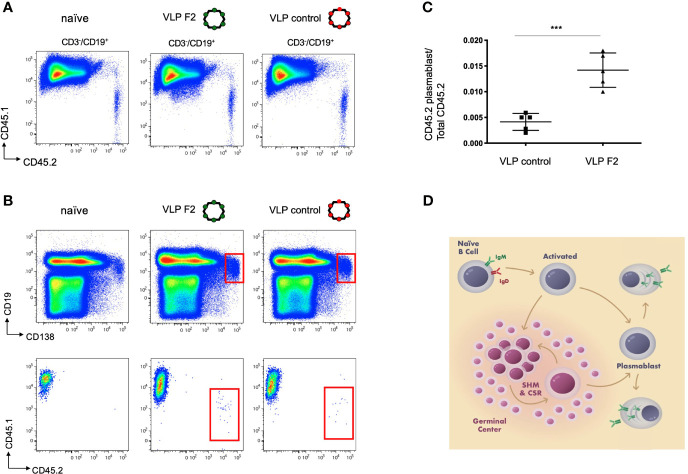
Selective IGHV1-2*02 B cell lineage expansion using the engineered vaccine immunogen VLP F2. **(A)** CD45.1^+/+^ mice received IGHV1-2*02 IgM B cells (1x10^6^ B cells per recipient) and were then either unimmunized, immunized with control VLP or immunized with VLP F2. CD45.1 *vs* CD45.2 reactivity is shown for CD3^-^/CD19^+^ B cells in each group. **(B)** CD45.1 *vs* CD45.2 reactivity was then evaluated within the plasmablast gate (CD19^int^/CD138^+^). **(C)** IGHV1-2*02 B cells expanded to plasmablasts in animals immunized with control VLP or VLP F2 (mean ± SD, n = 5 per group, standardized as the proportion of the total IgM IGHV1-2*02 B cells transferred to each recipient animal ***P < 0.001, Student’s T test). **(D)** Extrafollicular and follicular pathways for B cell activation and expansion following immunization, starting from the naïve IgM BCR stage through to differentiation to plasmablasts and plasma cells.

Our *in vitro* BCR signaling indicated that VLP-F2 was selective for IGHV1-2*02 BCRs (**Figures 2**, **3**). Because of their multivalent structures, and capacity to crosslink and activate BCRs, selected VLPs can elicit high titer antibodies responses against the selected peptide (72, 81, 82). To evaluate whether this V_H_-domain specificity translated into a capacity to selectively prime IGHV1-2*02 BCRs when present within a competing polyclonal germline repertoire, we generated a humanized vaccine model in which transgenic CD45.2^+/+^ IGHV1-2*02 B cells were diluted into recipient CD45.1^+/+^ C57Bl/6 mice, as we have performed previously (31). In this case, we first purified IgM B cells from IGHV1-2*02 HC2 mice, transgenic animals bearing the HC2 locus which enables restriction to a single V_H_ gene but simultaneous unconstrained human-like diversity in CDRH3 (31, 32, 53). We then transferred the CD45.2^+/+^ IGHV1-2*02 B cells into the bloodstream of CD45.1^+/+^recipients and monitored B cell expansion of the IGHV1-2*02 lineages within the spleen after intraperitoneal immunization (**Figure 4**) (31). Given that IGHV1-2*02 shows broad utility in both extrafollicular and follicular B cell responses (15, 53, 83), we sought to define whether VLP-F2 specificity for the IGHV1-2*02 domain would drive the differentiation of IGHV1-2*02 B cells into plasmablasts, a developmental stage that supports humoral output through both extrafollicular and follicular B cell pathways (84–86) (**Figure 4**). Compared to immunization with a VLP bearing an irrelevant peptide, we found that VLP-F2 enriched for IGHV1-2*02 B cell differentiation into plasmablasts, identified in mice as CD3^-^/CD19^int^/CD138^+^ (87). To account for IGHV1-2*02 B cell transfer efficiency, CD3^-^/CD19^int^/CD138^+^/CD45.2^+^ frequency was standardized to the total proportion of CD45.2^+/+^ B cells present in the spleen.

## Discussion

Deployment of shared features underscores reproducible broadly neutralizing antibody responses against microbial pathogens. We present methodology for generating virus-like-particle (VLP) vaccines that expand targeted human B cell lineages *via* engineering antigen specificity to the gene-encoded features of the germline antibody binding site. Antibody V gene-selective B cell priming has the potential to broadly augment antibody output from any public/V gene-selective response, such as against influenza virus, HCV, HBV, HIV, SARS-CoV-2, yellow fever virus, or the malaria parasite *Plasmodium falciparum* (13–22, 24–28, 88–92). While these antibody-target solutions can be immunologically recessive (and thus resistant to expansion by traditional vaccines), we have previously shown that germline-encoded affinity for cognate antigen can provide natural reproducible substrate for pathway-amplifying normally subdominant human influenza bnAbs (31, 32).

Compared to VLP displaying irrelevant peptide, we found that VLP-F2 enriched for the differentiation of naïve IgM IGHV1-2*02 B cells into plasmablasts, a stage of B cell expansion that is supported by both extrafollicular and follicular input (84–86). This provides experimental proof of concept for V_H_-biased B cell priming within an otherwise highly diverse germline antibody repertoire. Similar approaches have deployed adoptive transfer of monoclonal BCRs which are then activated by structure-based germline stimulating vaccines (37–39, 46, 70). By contrast, the IGHV1-2*02 B cells in our system were polyclonal, bearing unconstrained human-like diversity in their hypervariable CDRH3 loops (31, 53), which are the principal source of BCR diversity (4–6). Thus a key difference was to broadly prime human B cell lineages independent of their hypervariable features. While we cannot not rule out additional selection of CDRH3 structures in the response, a critical aspect of our approach was engineering VLP-affinity against the gene-encoded features of the BCR. This was enabled by the chimeric LK constructs, which facilitated the identification of VLP specificity to the IGHV1-2*02 domain. This specificity then manifested as a capacity to selectively trigger IGHV1-2*02 BCRs *in vitro* and selectively prime and expand polyclonal IGHV1-2*02 B cell lineages *in vivo*. Given the functional utility of IGHV1-2*02 in both follicular anti-viral responses and in extrafollicular anti-bacterial responses (15, 48, 53, 83), we suggest that V_H_-dependent B cell expansion could serve to intensify such activities, and could be conceivably serve as a directed activation step for any V_H_-gene dependent antibody response.

Another difference from current germline stimulating vaccine concepts is that our method does not rely on structure-based information for immunogen design, which can constrain experimental work on a few well-described B cell development pathways (37–39, 46, 70). Notably, we isolated VLPs bearing selective affinity for each LK selector antibody, suggesting that directed evolution on the VLP library harbors the potential to engage whichever BCR. Thus while we focused on specificity for the IGHV1-2*02 domain as proof of concept, our method could provide broad utility in engaging and expanding any BCR target of interest.

## Data Availability Statement

The original contributions presented in the study are included in the article/**Supplementary Material**. Further inquiries can be directed to the corresponding authors.

## Ethics Statement

The animal study was reviewed and approved by MGH Animal Committee, IACUC protocol 2014N000252.

## Author Contributions

LR, AY, JP, DP, BC, and DL designed the research studies. LR, AY, JP, VO, PD, and AM performed the research. RB, DR, and NL provided the transgenic mice. LR, AY, JP, DP, BC, and DL analyzed the data and wrote the paper. All authors contributed to the article and approved the submitted version.

## Funding

This work was supported by NIH funding to D.L. and B.C. (R01AI124378, R01AI137057, DP2DA042422, R01AI153098, R01AI155447), the Harvard University Milton Award, and the Gilead Research Scholars Program. The funder was not involved in the study design, collection, analysis, interpretation of data, the writing of this article or the decision to submit it for publication. JP was supported by NIH grant T32-AI007538. We thank the members of the Lingwood and Chackerian labs for helpful discussion and technical assistance.

## Conflict of Interest

BC and DP have an equity stake in Flagship Laboratories 72. RMB, DR and NL were employed by Bristol Myers Squibb.

The remaining authors declare that the research was conducted in the absence of any commercial or financial relationships that could be construed as a potential conflict of interest.

## Publisher’s Note

All claims expressed in this article are solely those of the authors and do not necessarily represent those of their affiliated organizations, or those of the publisher, the editors and the reviewers. Any product that may be evaluated in this article, or claim that may be made by its manufacturer, is not guaranteed or endorsed by the publisher.
