# Supplementary figures and images for: Engineering an Antibody V Gene-Selective Vaccine
Source: Front Immunol. 2021 Sep 9;12:730471. doi: 10.3389/fimmu.2021.730471 (PMC8459710; doi:10.3389/fimmu.2021.730471)

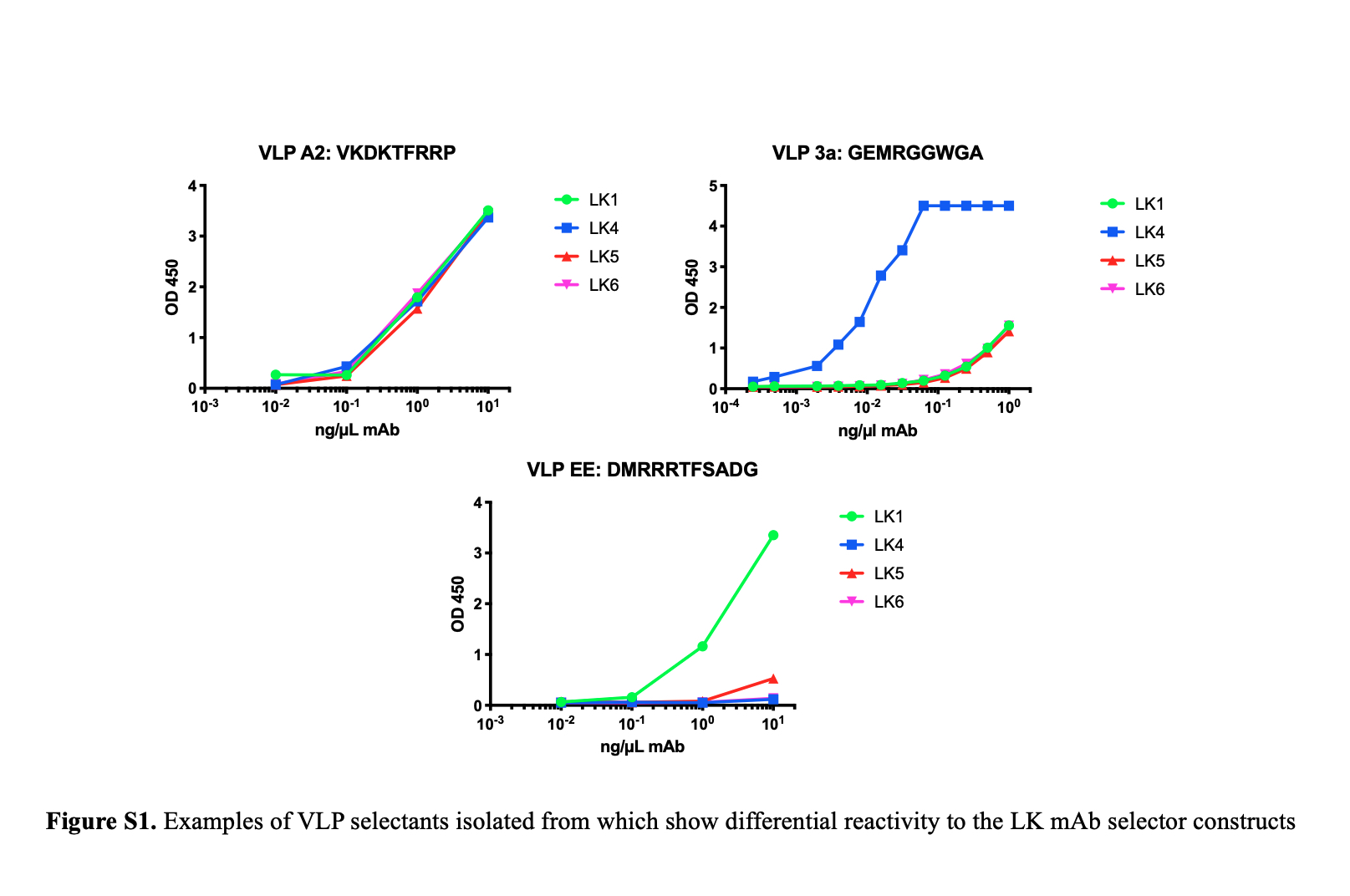

Supplement: Supplementary file 1 [file Image_1.jpeg]
